# Workshop Report: Systems Genetics of Neurodegenerative Disease, a Summer School in Systems Medicine, 25th August−1st September 2017

**DOI:** 10.3389/fgene.2019.00029

**Published:** 2019-02-05

**Authors:** Rupert W. Overall, Rudi Balling, Gerd Kempermann, Robert W Williams

**Affiliations:** ^1^Center for Regenerative Therapies Dresden, Technische Universität Dresden, Dresden, Germany; ^2^German Center for Neurodegenerative Diseases (DZNE), Dresden, Germany; ^3^Luxembourg Centre for Systems Biomedicine, University of Luxembourg, Esch-sur-Alzette, Luxembourg; ^4^Department of Genetics, Genomics and Informatics, University of Tennessee Health Science Center, Memphis, TN, United States

**Keywords:** training workshop, neurodegeneration, systems biology, genomics, data resources, data sharing

## Introduction

Systems genetics is a relatively new field that has developed with the rise of genomic big data and other -omics. Systems genetics attempts to relate DNA variants to gene expression and gene function, to physiological phenotypes and often to disease risk. The methods from this subdiscipline of systems biology are finding their way into mainstream biology and medicine and are thus no longer only the domain of statistical genetics. The awareness of how such methods can be used, however, is not widespread among experimental biologists and clinicians. We held a summer school, in August 2017^1^, with the aim of training early-career researchers and clinicians in the use of systems genetics tools and resources. This school was organized in order to address gaps between the fields of classical genetics, clinical research, and bioinformatics—where tools to analyze big data are created. In the post-genome age, there are now many well-curated data sets covering genome sequence, gene expression, interactions between genes and gene products, as well as genetically-modulated phenotypes and diseases. Most data sets however, despite being available in public repositories, lie unused. This is largely due to a lack of awareness and appropriate training among a large potential community of users who are best placed to apply the resources to clinical problems. In order to help bring these valuable data and the research questions closer, we trained young clinical researchers in ways to integrate the wealth of publicly available data in their research.

The focus of the summer school was on neurodegenerative diseases that typically exhibit complex genetic control. Worldwide aging populations mean there are increasingly more cases of neurodegenerative disease. The boom in genetic and genomic data collected over the last decade also means that there are now many resources available that are generally and specifically relevant to the study of neurodegeneration. The complex genetics of many of these disorders, such as Alzheimer's and Parkinson's diseases, requires a systems-level approach. The next generation of clinicians and clinical researchers must become comfortable with next-generation techniques if we are to put the already-existing rich data reserves to best use.

From a worldwide call for participants, we selected 25 PhD students and postdoctoral researchers to spend a week on an idyllic island in the Chiemsee in Bavaria, Germany. This small island, Frauenchiemsee, is dominated by an eighth century abbey (Abtei Frauenwörth), still home to a community of Benedictine nuns, which was the venue of the summer school. The participant trainees were joined by the four authors and four further internationally-renowned speakers; Professor Karl Broman (University of Wisconsin, Madison, WI, USA), Professor Rejko Krüger (Luxembourg Centre for Systems Biomedicine, Luxembourg), Dr. Jeremy Miller (Allen Institute for Brain Science, Seattle, WA, USA), and Dr. Victoria Perreau (Florey Institute, Melbourne, Australia). Despite the relatively small group size, the attendees of the summer school covered a broad spectrum of diversity with participants coming from eight different countries (Germany, USA, Australia, Luxembourg, the Netherlands, Slovenia, Sweden, and India). The 13 women and 12 men ranged from backgrounds in mathematics, bioinformatics, molecular biology, genetics, experimental neuroscience among others and included practicing clinicians. Successful participants were selected based on the quality of their applications which encompassed not only academic history and questions about current research, but also brief paragraphs of motivation and self-assessment. Key selection criteria were enthusiasm for genetics and systems biology and how much of an impact the school might make on their future research. Participants were not required to bring any specialist knowledge such as specific programming skills or clinical experience, although the selection process ensured that a range of expertise was present in each of the groups with the understanding that lateral transfer of knowledge between peers would be a key learning resource.

The innovative structure of the summer school pushes the traditional one-to-many seminar style into the background and highlights practical exploration of the data resources with close interaction between speakers/mentors and trainees, as well as a significant amount of peer-to-peer transfer of skills and information. Many of the participants were experts in their own right and the boundary between teacher and student often became blurred. The hands-on learning style of this format is centered around research projects carried out by teams of 5 participants each. The teams were tasked with refining their question in the context of available resources, constructing a novel analysis and writing up the results as a draft manuscript which was peer reviewed by the whole group.

The program began on the Friday of the arrival (25th August 2017), after a welcome dinner, with a lecture on *Systems Biomedicine* by Prof Balling, discussing the role of systems biology in the future of medicine. The next morning was filled with an in-depth workshop on *Genetic Resources for Neuroscience* by Prof Williams, focusing on the rich GeneNetwork resource^2^ followed after lunch by a lecture on *Genetic Stratification and Deep Phenotyping for Precision Medicine Approaches in Parkinson's Disease* by Prof Krüger. After this the participants split up into their five project teams and discussed, with guidance from the speakers/mentors, the question which would be the basis of their hands-on research project during the week. Day 3 of the summer school began with a morning workshop by Dr. Miller describing the *Allen Institute Resources* and how they can be applied. In the afternoon the teams presented their research topics and gained early-stage feedback from the whole group. This was followed by an evening lecture on *Genetic analysis of high-throughput phenotypes: challenges and opportunities* from Prof Broman, a primer on reproducible research in quantitative genetics^3^ Another workshop on *Using Genome Browsers and Network Tools* from Dr. Perreau gave a hands-on introduction of the many tools available (to be detailed in an accompanying manuscript) and rounded off the participants' toolbox which they were already applying to their research problems. After an afternoon of research downloading public datasets, scanning the literature and constructing analysis workflows using web tools as well as offline software such as R/Bioconductor, the final evening lecture was given by Prof. Kempermann on *Concepts in Systems Medicine* with a focus on knowledge management in the age of big data.

From this point, the formal part of the summer school was over and the remaining 3 days were entirely given over to research. This unique structure of our format gives participants real hands-on experience with applying the tools and resources they have just learnt and allows them to see the usefulness of these resources first-hand. Day 5 of the school was a pure research day, as was the morning of day 6 during which the teams also prepared their draft manuscripts. During these days, as for all times outside the formal lecture program, the study rooms with internet and stationery resources were freely available to the participants. The teams self-organized their research time and typically worked throughout the day and late into the evening. Outside mealtimes, coffee was of course provided regularly to fuel this intense activity! In the afternoon of the 6th day each team presented a summary of their project to the whole group. The 15-min formal presentations were impressive in their completeness with a well-structured flow and novel, interesting results being the norm. This after only 4 days of (albeit extremely intensive) research. On the 7th and final day there was a frantic rush to complete the project drafts which had to be submitted by midday. The submissions were again impressive and reflected the hard and focused work of the preceding week. Project research included the identification of transcriptional markers of disease, alterations in cell type diversity, unexpected similarities between expression profiles between diseases, diseases sub-type classification and molecular characterization of disease-specific pathways. The speakers/mentors reviewed all drafts after lunch, and each of the participants also received one draft from a different team to review. In the afternoon an innovative interactive peer review session took place. In this face-to-face review format the five team members/manuscript authors take their seats at the front of the group together with the five participants from other teams who reviewed their manuscript. The reviewers pose their questions and concerns and the authors have the opportunity to directly respond in a 20-min intense discussion. The issues raised during this session were invaluable to the teams for the development of their research topic into an actual peer-reviewed manuscript over the coming year. We strongly encourage all teams to pursue this, and we have proven in the past that such a format can work well. Six manuscripts from a previous summer school were published following successful peer review in a Frontiers Research Topic in 2015 (Overall et al., 2015). This is the first time, to our knowledge, that a training workshop has given rise to such tangible results.

The manuscripts from our previous workshop provide an idea of the scope and scale of project that can be addressed in a summer school format. We are aware of many other similar training and research events, ranging from “top down” educational workshops to “bottom up” hackathons and project-oriented brainstorming events. A number of these also incorporate active research on projects and some have even driven manuscript preparation. Our still-evolving summer school format has aimed to incorporate the best aspects of such events to balance the transfer of knowledge between all participants—be they teacher or student. The production of manuscripts is not a primary goal of the summer school, but it is a reflection of the enthusiasm of the participants that the ideas brought together during the initial week were so effectively developed. Although the published manuscripts actually reflect a full year's worth of hard collaborative work, the ideas, structure and many results were already laid down and spelled out in the week of the summer school. This was partly made possible by the particular properties of the venue. Being an island, the participants were undistracted by travel, restaurant-seeking, group fragmentation or student-speaker stratification issues that so often plague traditional conferences and workshops. The mentors were always available and shared all meals together with the participants. The free 24-h access to study rooms meant that the groups could self-organize their time—slipping in a coffee break or a swim in the lake while datasets were downloading. Discussions often continued over beer late into the evening. The groups rapidly identified the expertise and experience present among the participants and mentors, and tasks were dynamically assigned accordingly. Inter-group collaboration was common and missing skills were taught and learnt as needed. The groups laid down a question and a research plan over the first 2 days and fleshed this out with results in a focused program to build the drafts in real time using collaborative authoring tools. The manuscript sketches grew rapidly and in most cases the outline for the following year of work was already in place by the end of the week. Continued collaboration, despite the authors being spread across the globe, has seen the manuscripts mature. At the time of writing, the Research Topic associated with the current summer school^4^ is still open—we are eager to see if some manuscripts will also be accepted from the drafts begun in that week in August 2017.

The secondary goal of publications aside, the meeting as a training opportunity was a success, with many participants excited to integrate the new skills into their research programs. We hope that the knowledge and way of approaching genetic research questions inspired in the participants will also rub off on their colleagues, helping to make even more researchers aware of the wealth and utility of the available resources. We look forward to seeing what effect this systems genetics training will have on developments in the field, and we hope to host more, similar training opportunities in the future. In addition, we believe that the workshop format we have developed could be applicable to a wide range of research fields and we hope that our summer school will provide a template for other similar events.

## Author Contributions

All authors listed were co-organizers of the summer school, contributed to the manuscript and approved it for publication.

### Conflict of Interest Statement

The authors declare that the research was conducted in the absence of any commercial or financial relationships that could be construed as a potential conflict of interest.

## Footnotes

^1^http://sysgenschool.org/sgn2017

^2^http://www.genenetwork.org

^3^https://www.biostat.wisc.edu/~kbroman/presentations/SGN2017/sgn2017.pdf.

^4^https://www.frontiersin.org/research-topics/6173/systems-genetics-of-neurodegenerative-disease
